# In-depth study on resonant tunneling for subwavelength imaging

**DOI:** 10.1038/s41598-018-33653-y

**Published:** 2018-10-19

**Authors:** Md. Anzan-Uz-Zaman, Kyungjun Song, EunJoong Lee, Shin Hur

**Affiliations:** 1 Department of Nature-Inspired Nano Convergence Systems, Korea Institute of Machinery and Materials, Daejeon, (34103) 156 Republic of Korea; 2 Nano-Mechatronics, University of Science and Technology, Daejeon, (34113) 217 Republic of Korea

## Abstract

We report new frequency bands for subwavelength imaging by using the resonant tunneling method which have not been explored previously. As per the existing theory of resonant tunneling, imaging frequency is limited for a certain number of crystals. However, after conducting an analytical analysis over a wide range of frequencies, we observed that higher frequencies do exist for subwavelength imaging. We verified this observation both numerically and experimentally. We extended our study to observe the effect of lattice periodicity on image resolution. By reducing periodicity during experiment, we achieved a resolution of λ/9.5 at the conventional region and λ/2.45 at the higher band region.

## Introduction

Abbe limit^1^, which is commonly known as the diffraction limit, is a natural constraint on image resolution that roughly implies that the maximum achievable resolution corresponds to half of the wavelength. To obtain a good-quality image, it is necessary to overcome this limit. Since Pendry’s successful work on perfect lens^2^, several methods have been proposed to overcome the diffraction limit in both the electromagnetic and acoustic wave regimes, such as the time reversal technique^3–6^, Brag Scattering^7–9^, superlens^10–24^, and hyperlens^25–30^. According to our ken, the best resolution reported for acoustic waves is λ/50^20^. The technique behind the lens was strong coupling of evanescent wave through Fabry‒Pérot (FP) resonance. As evanescent waves contain finer feature information of an object, by recovering them at the image plane by the lens, it was possible to obtain subwavelength imaging. However, in this method, the imaging frequency is dependent on lens thickness as the FP resonance is dependent on it. To reduce the dependency on lens thickness, zero-mass^19,31,32^ and Bloch-wave-based anisotropic resonant tunneling^33,34^ metamaterials have been anticipated. For the resonant tunneling case, both evanescent and propagating waves will be tunneled if the lens thickness is equal to the integer multiple of half of the Bloch wavelength. This implies that the imaging frequency can be altered without changing the lens thickness by modifying the micro-structure of the lens. However, it has been observed that the imaging frequency tends to decrease on increasing the diameter modulation factor, and the highest imaging frequency is obtained^33^ when the modulation factor is unity, which is in fact the FP resonance condition. As subwavelength imaging has potential application at higher frequency regime, an FP-based lens seems more suitable than resonant tunneling. However, the frequency of FP-based lens is also limited owing to the requirement that the wavelength should be much higher than the lattice periodicity and hole diameter^20^. Besides this, except refs^14,21^, most previous studies in the acoustic regime were based on low frequency. Thus, we studied resonant tunneling and discovered that higher bands of frequency exist for it, which was not possible according to previous works^33,34^. Moreover, unlike an FP-based lens, the wavelength of a resonant tunneling lens need not be very higher than the lattice periodicity and hole diameter. Therefore, we hope that our work will open new avenues for practical application of subwavelength imaging.

## Results

### Analytical Study

**Figure 1:**
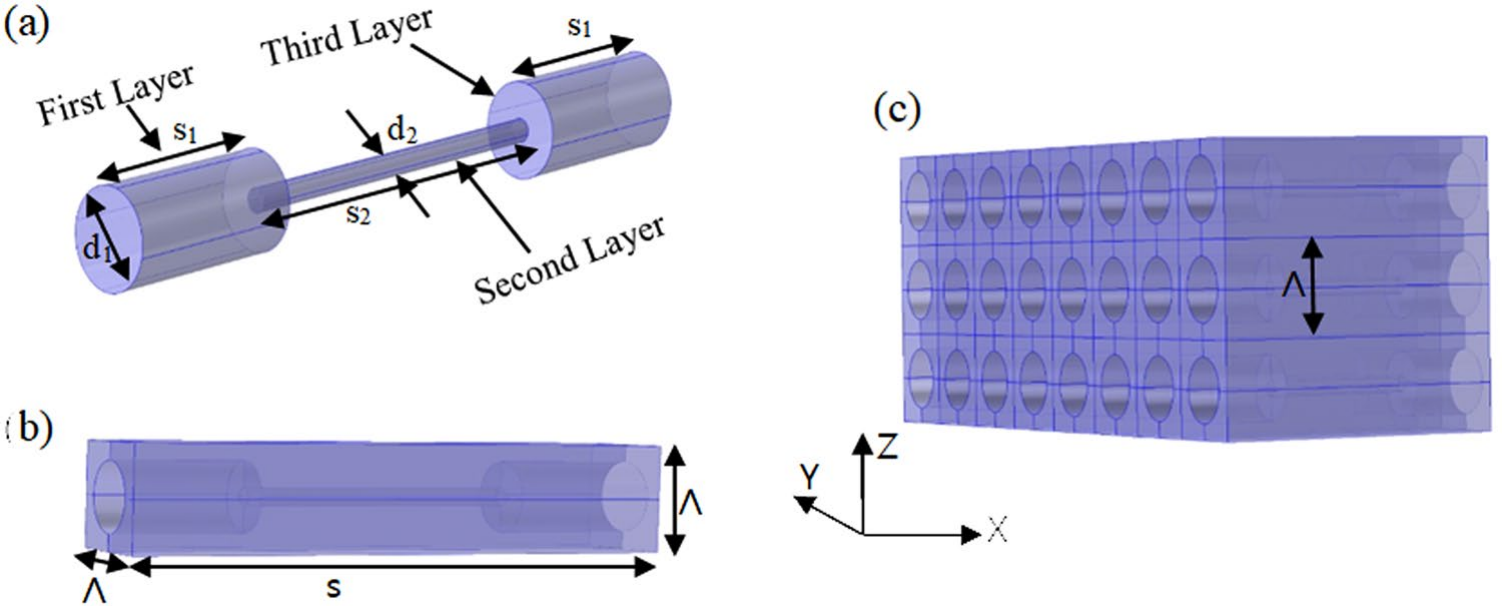
(**a**) Three-layered single hole with diameter modulation; **(b)** Single hole into a unit cell. **(c)** Holey- structured single crystal.

We used a holey structured metamaterial introduced by H. Su *et al*.^33^, in a periodic array, which is shown in Fig. 1. Here, the unit cell diameter of a crystal has been modulated along the X direction and its length, s = 2s_1_ + s_2,_ is shown in Fig. 1a. It should be noted that we have used s_2_ = 2s_1_. The diameter of the first and third layers of the single hole are d_1_ and that of the second layer is d_2_. The diameter modulation factor is δ = d_1_/d_2_ = 4. The lattice periodicity of the crystal is denoted by Λ. The holes are filled with air, and the boundary of the holes are made of hard material for acoustic waves, except at the opening and end. The three layer (i = 1, 2, 3) effective mass density of a unit cell can be expressed in tensor format as^35^

1 $$\begin{array}{rcl}{\tilde{\rho }}^{(i)} & = & diag[{\rho }_{i}^{x},{\rho }_{i}^{y},{\rho }_{i}^{z}],\\ {\rm{with}}\,{\rho }_{i}^{x} & = & {\rho }_{air}\frac{{\Lambda }^{2}}{(hole\,area)}={\rho }_{0}\frac{{\rm{4}}{\Lambda }^{2}}{\pi {{d}_{i}}^{2}},\,{\rho }_{i}^{y}={\rho }_{i}^{z}=\infty \end{array}$$

Here, ${{\rm{\rho }}}_{{\rm{air}}}={{\rm{\rho }}}_{0}=1.25\,{\mathrm{kg}/{\rm{m}}}^{3}$ and d_i_ = diameter of the respective layer.

If the number of crystals be N, the transmission equation can be given as^33,34^

2 $$T(\omega ,{k}_{\Vert })=\frac{{z}_{0}}{{z}_{0}\,\cos (Nqs)-\frac{({T}_{1}+{T}_{2}{z}_{0}^{2})\,\sin (Nqs)}{2\,\sin (qs)}}$$

where,

3 $${T}_{1}=i{\rho }_{2}^{x}{c}_{0}[\frac{1}{{\delta }^{2}}cos{k}_{0}{s}_{2}\,\sin \,2{k}_{0}{s}_{1}+\,\sin \,{k}_{0}{s}_{2}\times (co{s}^{2}{k}_{0}{s}_{1}-\frac{1}{{\delta }^{4}}si{n}^{2}{k}_{0}{s}_{1})]$$

4 $${T}_{2}=\frac{i}{{\rho }_{2}^{x}{c}_{0}}[{\delta }^{2}cos\,{k}_{0}{s}_{2}\,\sin \,2{k}_{0}{s}_{1}+\,\sin \,{k}_{0}{s}_{2}\times (co{s}^{2}{k}_{0}{s}_{1}-{\delta }^{4}si{n}^{2}{k}_{0}{s}_{1})]$$

5 $${z}_{0}=\frac{{\rho }_{0}\omega }{\sqrt{{k}_{0}^{2}-{k}_{\Vert }^{2}}};$$

q = wavenumber of the Bloch Wave along the X-direction, *c*_0_ = sound velocity in air = 343 m/s,

incident wave number, ${k}_{0}=\sqrt{{k}_{x}^{2}+{k}_{y}^{2}+{k}_{z}^{2}}=2\pi /\lambda ,\,{k}_{||}=\sqrt{{k}_{y}^{2}+{k}_{z}^{2}}$

The dispersion relation for the propagating Bloch wave along the X-direction can be determined as^33^

6 $$\cos \,qs=cos\,{k}_{0}s-\frac{1}{2}{(\delta -\frac{1}{\delta })}^{2}sin\,{k}_{0}{s}_{2}\,\sin \,2{k}_{0}{s}_{1}$$

We know at the resonant tunneling condition, 100% transmission will occur for both propagating and evanescent waves^34^, i.e. $|T|=1$.

From equation (2), we can see that tunneling can be achieved by satisfying the following condition^34^

7 $${\rm{Nqs}}=m{\rm{\pi }},\,{\rm{m}}=1,2,3\ldots \ldots \ldots \ldots \ldots \ldots \ldots \ldots .,\,{\rm{N}}-1$$

From equations (2) and (7), we realize that for tunneling, the minimum number of crystals (N) required are two. The diameters are taken as d_1_ = 4d_2_ = 8 mm. By putting the value of *δ* and replacing s_1_ and s_2_ in terms of s, we can simplify the dispersion equation (6) for N = 2 as follows:

8 $$\cos \,{{\rm{k}}}_{0}{\rm{s}}=\frac{225}{289}$$

**Figure 2:**
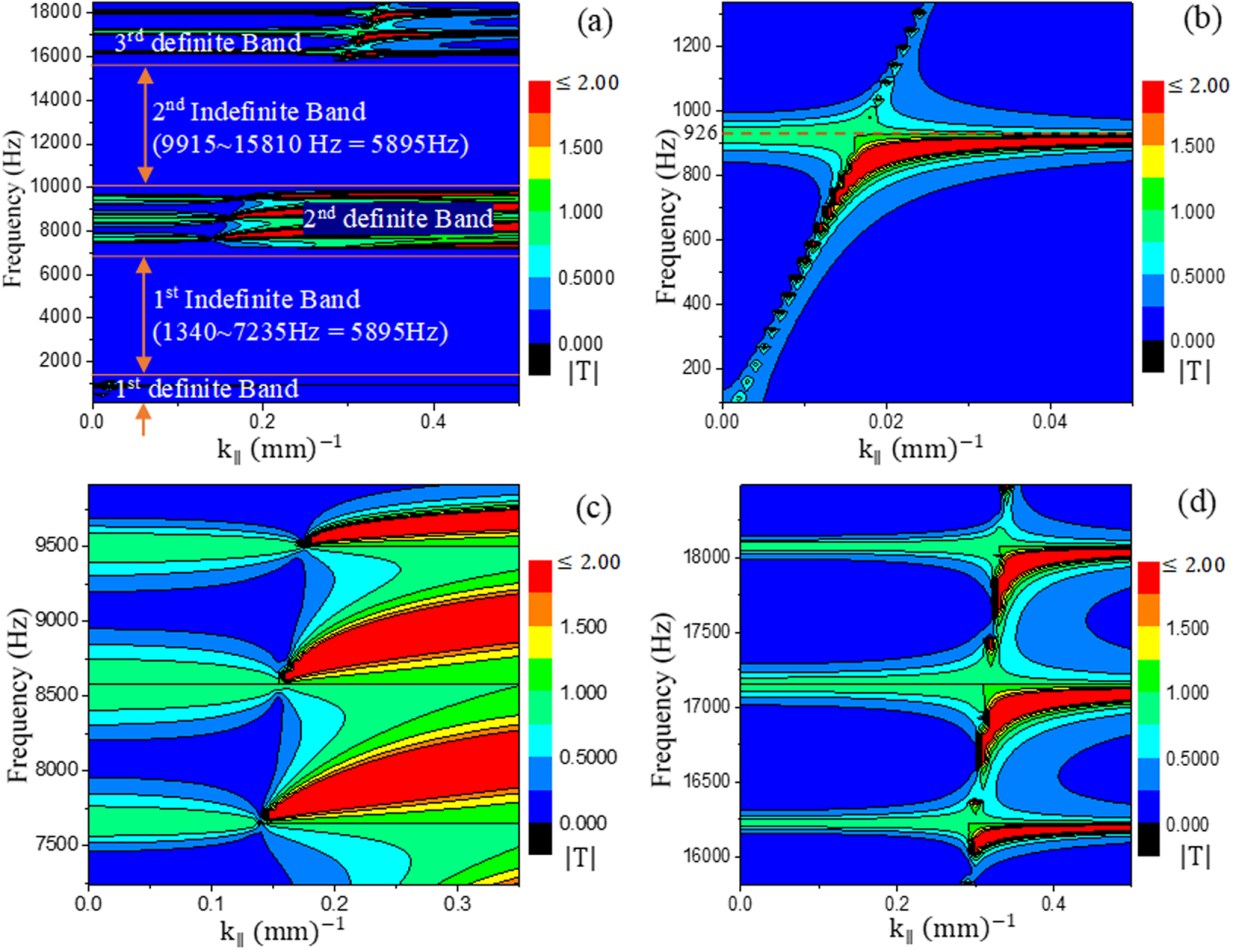
(**a**) Contour plot of transmission co-efficent amplitudes |T| for a wide band frequency ranging from 100 Hz‒18490 Hz with respect to different parallel wavenumbers. **(b)** Amplified view of the contour plot for the first definite region of the frequency band up to 1335 Hz. **(c)** Amplified view of the second definite region for a bandwidth (BW) of 7240‒9910 Hz (=2670 Hz). **(d)** Amplified view of the third definite region for a BW of 15815‒18485 Hz (=2670 Hz).

For s = 40 mm^33^, we can deduce the tunneling frequency from equation (8) as f = 926 Hz. It should be noted here that this is the highest and only frequency for the given number of crystals, N = 2, as per equation (7). However, we have analytically examined all the values of qs from 100 Hz‒18490 Hz according to equation (6) and found that for certain bands of frequency, its value becomes indefinite as −1 > cos qs > 1 and is repetitive in nature (see Fig. 2a). For example, for our current parameters, until 1335 Hz, the value of qs is definite as $-\,1\le \,\cos \,qs\le 1$. Then, for a bandwidth (BW) of 1340‒7235 Hz (=5895 Hz), it becomes indefinite. Again, the value of qs becomes definite for a BW of 7240‒9910 Hz (=2670 Hz), and then, it becomes indefinite again for a BW of 9915‒15810 Hz (=5895 Hz), and this process continues. We have determined the transmission co-efficient for a periodicity of Λ = 10 mm^33^ and found some interesting results at higher definite band regions which indicated that bands of frequencies exist for tunneling, as shown in Fig. 2.

From Fig. 2b, we see the first tunneling frequency (926 Hz) in the first definite region which was determined by equation (8). From Fig. 2c,d, we can see that at the second and third definite regions, bands of frequencies exist for imaging. Therefore, analytically, we can realize frequencies higher than the first tunneling frequency of 926 Hz. These findings indicate that we can extend the subwavelength imaging to higher frequency regimes to detect tiny objects with a smaller feature size. It should also be noted that at the third definite region, the bands of tunneling frequencies are narrower than those at the second definite region.

**Figure 3:**
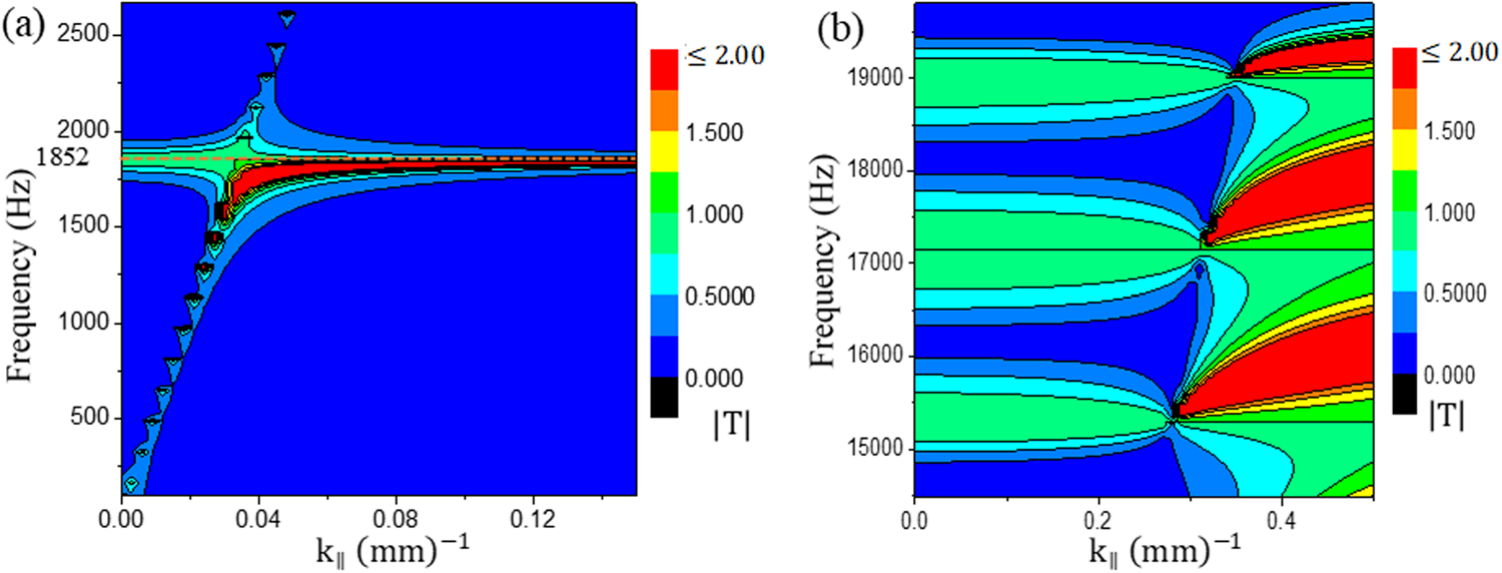
Contour plots for new crystal parameters with crystal thickness s = 20 mm. **(a**) |T| of the first definite region(0‒2670 Hz); **(b)** |T| of the second definte region (14480‒19820 Hz = 5340 Hz).

Inspired from the results mentioned above, we have designed a new lens with a crystal thickness of s = 2s_1_ + s_2_ = 20 mm with s_2_ = 2s_1_ = 10 mm, d_1_ = 4d_2_ = 4 mm, and Λ = 6 mm. We keep the total number of crystals as N = 2 which implies a lens thickness of 2 s = 40 mm. Equation (8) can still be applied to the current parameters of the lens for deducing the first tunneling frequency, and it should be 1852 Hz. During analytical computations, we have observed that decreasing the crystal thickness (s) by half, the definite and indefinite region of frequency has been doubled. By using a step size of 5 Hz in our calculation, we obtain a BW of 5340 Hz (except for the first definite region, which is 2670 Hz) and 11800 Hz for the definite and indefinite regions, respectively. The first definite region exists until 2670 Hz (Fig. 3a), which is half of the next definite region, 14480‒19820 Hz (=5340 Hz) (Fig. 3b). We have performed analytical calculations up to 21000 Hz which comprises only the first two definite regions of frequency. As tunneling is possible only in the definite regions, we have focused on these regions. The transmission characteristics are shown in Fig. 3.

From Fig. 3a, the first tunneling frequency can be observed at 1852 Hz. On the other hand, as shown in Fig. 3b, three bands of frequencies are observed for tunneling. At second band in Fig. 3b, we see a straight line is appeared slightly above 17000 Hz. This line corresponds to a special frequency which does not pertain for subwavelength imaging as no tunneling is possible for this frequency. Analytically this phenomenon can be understood. If we rewrite equation (6) for our current parameters, may be written as

9 $$\cos \,qs=cos\,{k}_{0}s-\frac{1}{4}{(\delta -\frac{1}{\delta })}^{2}(1-cos\,{k}_{0}s)\,$$

or,

10 $$\cos \,qs=1,\,for\,{\rm{s}}={\rm{n}}\lambda \,{\rm{or}},\,\lambda =s/n\,{\rm{where}}\,{\rm{n}}={\rm{any}}\,{\rm{integer}}\,{\rm{number}},$$

If cos *qs* = 1, equation (2) becomes indefinite because of the “sin(Nqs)/sin(qs)” term at the denominator. Therefore, $|T|={\rm{indefinite}}$ for any λ = s/n. For our case, if n = 1, tunneling will not be possible at λ = 20 mm, which corresponds to 17150 Hz. The fact can hardly be distinguished from Fig. 3(b) as it is a single frequency inside the tunneling band which has such non- tunneling characteristic. Therefore, it is necessary to mention them separately. We term these frequencies as prohibited frequencies.

On a different note but not unrelated to this topic, we were also interested to investigate the fact that what happens if we interchange the diameters of the layers such as 4d_1_ = d_2_ = 4 mm which means the narrow tunnel will be at the both end and wide tunnel at the middle of the unit cell. Although the modulation factor, δ (=d_1_/d_2_ = 1/4) has been reduced to 1/4, according to equation (6) the definite band and indefinite band size will be the same as ${(\delta -\frac{1}{\delta })}^{2}$ factor remains unchanged. From equations (2), (7) and (8), we understand the tunneling frequency at the first definite band will be the same as previous which is 1852 Hz. But, from equation (2), it is not possible to predict the case about the second definite band, as T_1_ and T_2_ has been changed due to interchange of diameters. However, we obtained the transmission behavior for both of the definite bands analytically (see Supplementary Information, Fig. S7) and found for second definite regions, there are no band for tunneling. But, interestingly there was one more tunneling band at the end of the first definite region. At our next work, we will investigate about it rigorously. The facts of tunneling at the first definite region and not tunneling at the second definite region has been demonstrated numerically and represented at the Supplementary Information (see Fig. S8).

### Numerical Analysis

**Figure 4:**
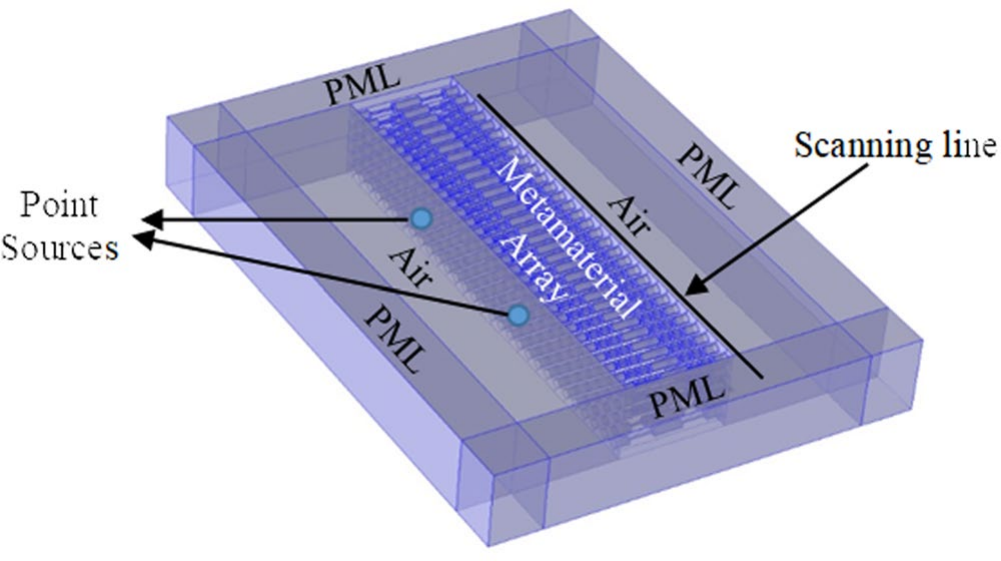
Simulation model for verifying subwavelength imaging for the proposed lenses. The scanning line and point sources (amplified view) are at the same height. Point sources are placed 1 mm in front of the lens and pressure amplitudes are obtained along the scanning line which is placed 1 mm behind the lens.

To corroborate our analytical findings, we have used a commercial multi-physics software COMSOL for numerical analysis. We have made the lens with an array of hole 30 × 5 along the (Y, Z) directions with a periodicity of Λ = 6 mm. All other parameters were identical to those of the previously developed lens: s = 2s_1_ + s_2_ = 20 mm, s_2_ = 2s_1_ = 10 mm, d_1_ = 4d_2_ = 4 mm. A perfectly matched layer (PML) having a thickness of 20 mm was used around the model. Only the air part was meshed to determine the transmission behavior. Acoustic Pressure, Frequency Domain (acpr) Physics was used in our simulation model. The model can be seen in Fig. 4.

**Figure 5:**
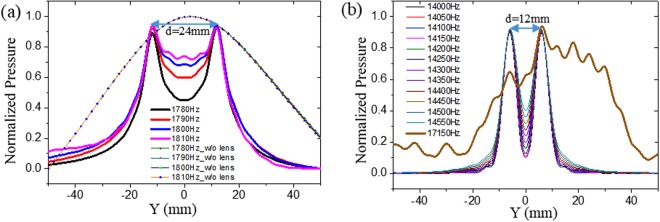
Simulation result: Normalized pressure amplitudes 1 mm behind the lens. **(a)** Frequencies at the first definite region for which subwavelength imaging was obtained. The distance between the sources (d) is 24 mm. Solid lines represent measurement with lens and dashed and dotted lines without lens. **(b)** A part of the second definite region. The distance between the sources (d) is 2Λ = 12 mm. The thick line represents the prohibited frequency at which no imaging was possible.

As shown in Fig. 4, the monopole point sources were taken as speakers and placed in front of the lens at a distance of 1 mm from it. The flow rate of the point source was 10 m^3^/s, and distance between them was 24 mm. The position of the scanning line was at the same height as the sources along the Z-direction. Scanning was performed 1 mm behind the lens along the Y direction. Subwavelength imaging can be realized at the frequencies shown in Fig. 5.

From Fig. 5a, it is evident that our crystal can detect point sources at a subwavelength distance (24 mm ≈ λ/8), whereas without lens, no sources can be detected. A small discrepancy was found between the theoretical imaging frequency (1852 Hz) and the best simulation frequency (1780 Hz). We will discuss about it at next at the chapter “Effect of periodicity on subwavelength imaging”.

From Fig. 3b, it can be seen that, theoretically, the first band of frequency at the second definite region for which imaging is possible ranges from 15000‒16000 Hz. To obtain subwavelength imaging in this region, we need to reduce the distance between the sources to approximately 10 mm. However, we noticed that imaging can be obtained until a distance of 2Λ, which is related to periodicity and will be discussed at next (see section “effect of periodicity on subwavelength imaging”). From Fig. 3b, a small band of frequency (approximately 300 Hz) is also observed at which no imaging occurs. However, we found some discrepancy between the theoretical first band of the second definite region (15000‒16000 Hz), as the simulation frequency for imaging has been found for a BW of 13400‒15300 Hz. Anyway, a portion of the imaging BW of 14000‒14550 Hz has been represented in Fig. 5b. The remaining frequencies in this BW can be found in Supplementary Information (Figs S1 and S2). During the simulation, we also observed that no imaging region exists between 15350 and 15750 Hz (400 Hz) which is similar to the theoretical prediction.

From Fig. 3b, we can see that the second band is the widest band among all the three bands and ranges from 16300‒18000 Hz. It should be noted that the prohibited frequency (17150 Hz) lies inside this band. During simulation, we also found that a wide BW of 15800‒18700 Hz for imaging, in addition to a narrow band (16950‒17350 Hz) of non-imaging frequencies around the prohibited frequency of 17150 Hz (See Figs S3 and S4). The non-imaging nature of the frequency 17150 Hz can be seen in Fig. 5b.

According to Fig. 3b, after a narrow band of no-imaging region, we should observe the third band which approximately ranges from 18300 to 19800 Hz. The gap between the second and third bands is almost 300 Hz. According to our simulation result, the gap was found to be 150 Hz, ranging from 18750 to 18900 Hz. In the simulation, imaging was observed in the third band from 18950 to 20000 Hz (See Fig. S5). Thus, from the above discussion, it can be said that with some inconsistencies, the simulation result coincide well with the theoretical model. The next section will describe the experiment conducted by the designed lens.

### Experimental Study

**Figure 6:**
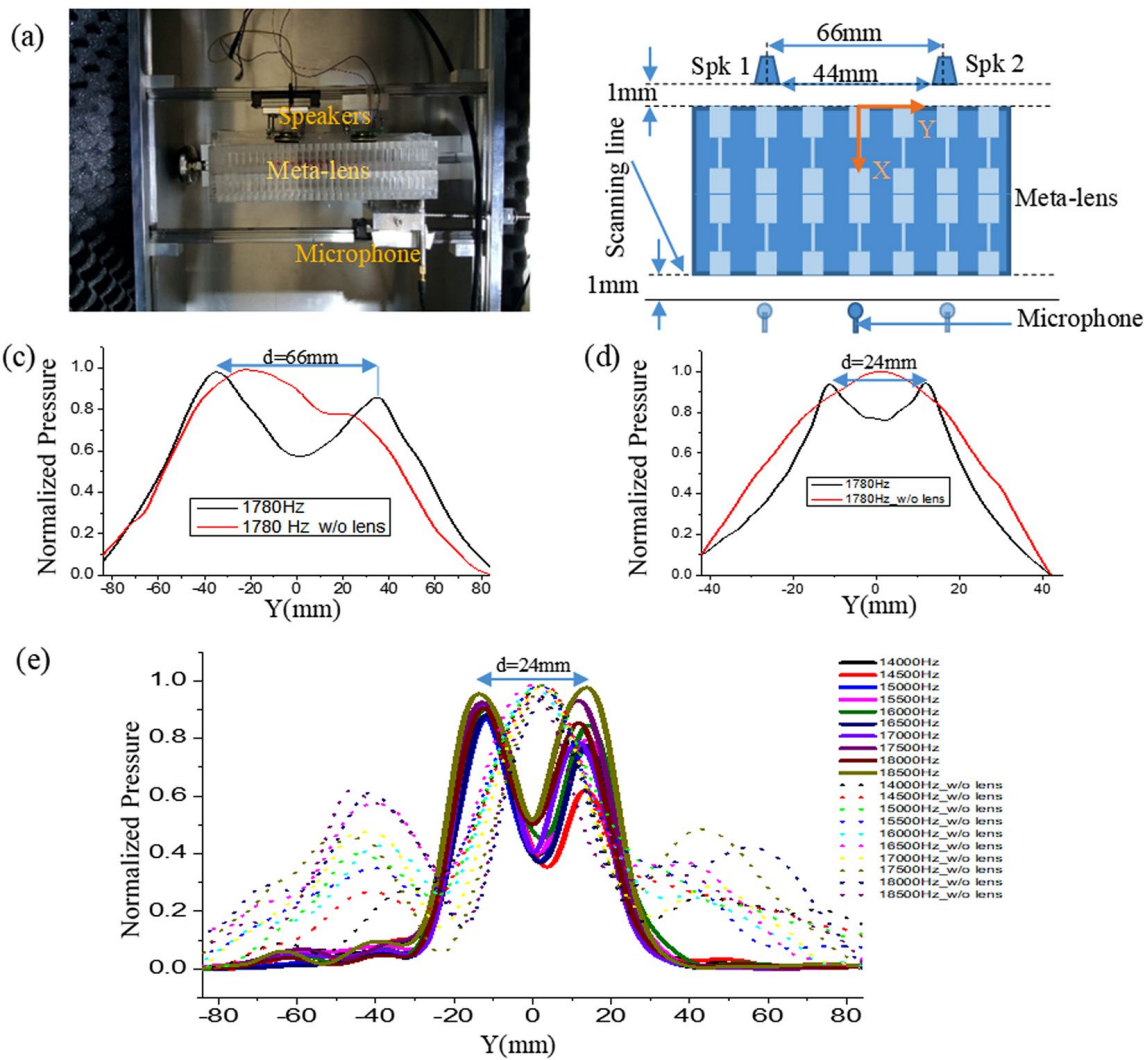
Here, d denotes the center to center distance between the speakers. **(a)** Experimental set-up for verifying subwavelength imaging. **(b)** Conceptual 2D set-up of the experiment. **(c)** Normalized pressure amplitudes at a distance 1 mm behind the lens where black line and red line represent the data with lens and without lens respectively. **(d)** Normalized pressure amplitudes at a distance 1 mm behind the lens with a step size 3 mm. **(e)** Normalized pressure amplitudes for the second definite region. Solid lines and dotted lines represent data with lens and without lens, respectively.

The metamaterial was prepared by mechanical machining of the Polymethyl methacrylate (PMMA), bars, which can be regarded as hard material for sound propagation with respect to air. As shown in Fig. 6a, the lens was prepared by placing two crystals together tightly. The speaker diameter was 22 mm and placed at a distance of 1 mm in front of the lens. The centre to centre distance between the two speakers was 66 mm. The microphone was placed at the same height as the centre of the speakers. The microphone was moved along the Y direction at a step size of 6 mm. The two sources separated by 66 mm (≈λ/3) can be easily discernible from Fig. 6c; however, without the meta-lens, it cannot be identified. Although we get the resolution beyond the diffraction limit, but still it is far away from the numerical result. This might stem from the air leakage due to direct coupling without any sealing process between one crystal and another. However, we overcome the problem using adhesive material to couple the crystals and 1.5 mm diameter MEMS speakers. The distance between the speakers was 24 mm. Now, as shown in Fig. 6d, we get higher resolution (≈λ/8) which is similar to the simulation result (Fig. 5a).

Next, for obtaining the image at the 2nd definite region, we used speakers with 22 mm diameter. We maintained a 2 mm distance between the two speakers which yielded a 24 mm center to center distance. As shown in Fig. 6e, the two speakers which were separated by only 2 mm can be easily resolved. This result provides us the experimental evidence that much higher frequencies exist for tunnelling, which is not governed by equation (7).

From Fig. 6e, it can be observed that all the three bands are covered in the 2nd definite region with a step size 500 Hz starting from 14000 to 18500 Hz. Because the no-imaging band is very narrow compared to our step size, no such frequency appeared in our graph. Although we have shown only the better imaging frequency region, in the experiment, we found imaging for a BW 13500–19000 Hz, which is approximately close to both theoretical and simulation results. Furthermore, Fig. S6 differentiates the imaging region from the non-imaging region. However, for without lens case at Fig. 6e, two small peaks were appeared around ± 40 mm distance. We assume this might be inherited from interference.

### Effect of Periodicity on Subwavelength Imaging

**Figure 7:**
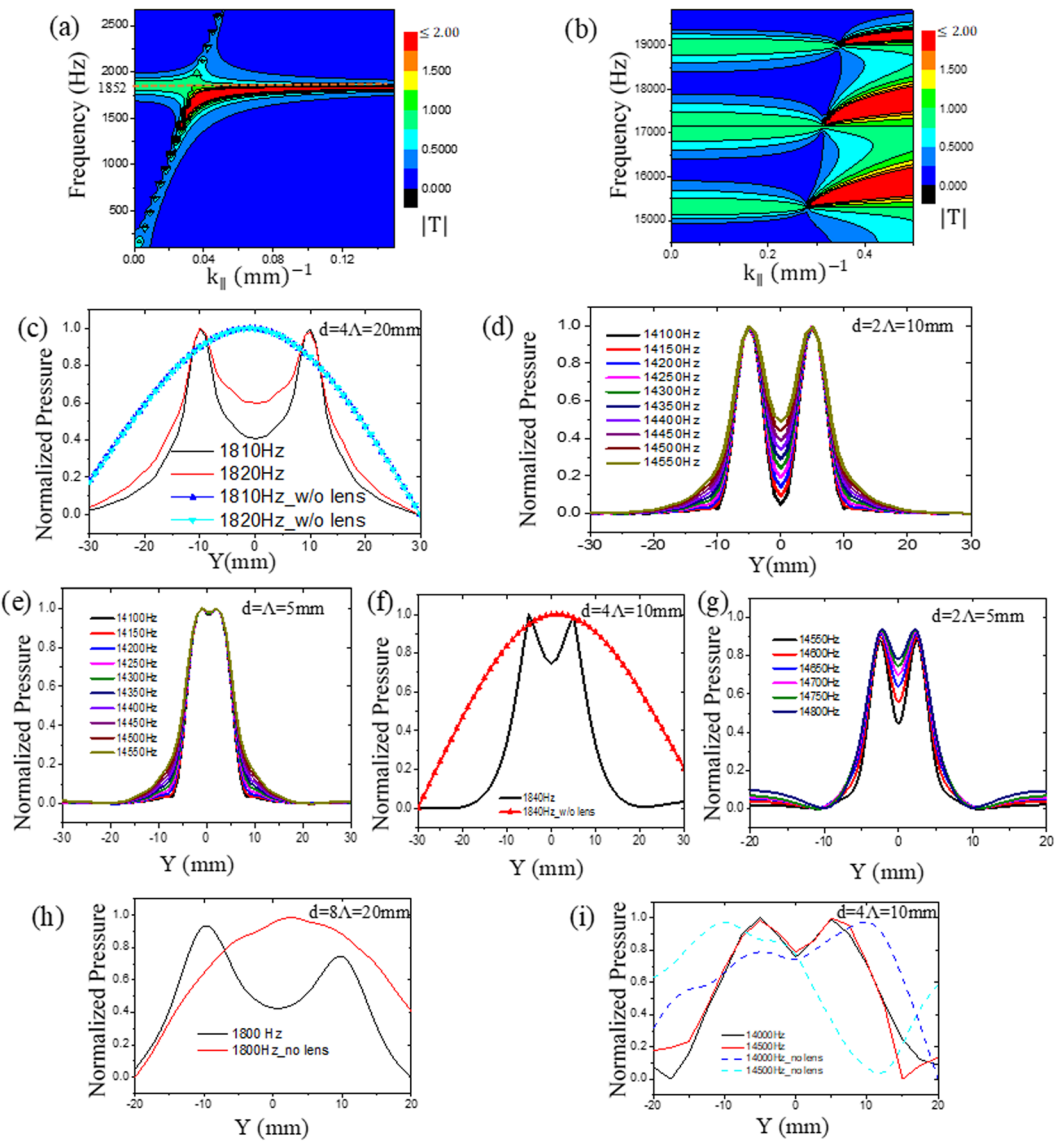
Here, d = distance between the sources. Contour plot of transmission co-efficient amplitudes |T| for a lens with 5 mm periodicity **(a)** for the first definite region; **(b)** for the second definite region; Numerical study: Normalized pressure amplitudes at a distance 1 mm behind the lens **(c)** for a lens with Λ = 5 mm and distance between the sources 20 mm; **(d)** for distance between the sources 10 mm (=2Λ) at 2nd definite region; **(e)** for distance between the sources 5 mm (=1Λ); **(f)** for a lens with Λ = 2.5 mm and distance between the sources 10 mm; **(g)** for distance between the sources 5 mm (=2Λ) at 2nd definite region; Experimental result with Λ = 2.5 mm: **(h)** at the first definite region **(i)** for distance between sources 10 mm (=4Λ) at 2nd definite region.

In the numerical analysis chapter, it was discussed that periodicity is the prevailing factor for obtaining subwavelength imaging, as the wavelength becomes comparable to it at a higher frequency region. Therefore, we decided to reduce the periodicity by keeping all the other parameters same. We reduced periodicity from 6 mm to 5 mm and conducted the contour plot of transmission amplitudes, which is depicted in Fig. 7. Though we have reduced the periodicity (Λ), according to equation (7), for s = 20 mm, our first tunnelling frequency remains the same 1852 Hz as with the periodicity 6 mm. However, the tunnelling frequency according to the simulation is 1810 Hz (see Fig. 7c). From Fig. 7b, we can observe the band regions have been changed slightly than from Fig. 3b as effective masses (see equation (1)) of the respective layers have been changed, which have effect on T_1_ and T_2,_ affected the transmission behaviour. From Figs 5b and 7d, we observe that the image can be generated until the 2Λ distance. However, as Λ has been decreased to 5 mm, the object can be detected for a 10-mm distance, which is less than the previous lens with 6 mm periodicity. Figure 7e represents the data for 1Λ distance between the speakers; it is realised that it is difficult to produce a reliable image with this distance.

Next, we decreased the periodicity (Λ) further to 2.5 mm with d_1_ = 2 mm and d_2_ = 0.5 mm. From equation (1), it can be realized that the effective mass of the respective layer would remain same with the crystal with periodicity 5 mm as Λ/d_i_ remains the same. Thus, analytically, there should not be any difference in the transmission behaviour between the two crystals. However, from Fig. 7f,h, the first tunnelling frequencies obtained numerically and experimentally were 1840 Hz and 1800 Hz, respectively. This is closer to the theoretical frequency 1852(Hz). From this point of view, by observing Figs 5a, and 7(c,f,h), we may conclude that by reducing the periodicity, the tunnelling frequency tends to approach towards the theoretical frequency. By reducing the periodicity and hole diameters, we can image at the first definite region down to 20 mm or λ/9.5 (see Fig. 7h), which is much better than earlier (Fig. 6c).

The numerical result at Fig. 7g shows that for a 2Λ(=5 mm) separation between the speaker, the new lens can produce the image similar to the previous lenses at the second definite region. However, as the periodicity reduced, for a frequency 14500 Hz, the resolution reduces to approximately λ/4.7. From Fig. 7i, up to 4Λ(=10 mm) resolution was obtained experimentally by the lens which is also below the diffraction limit and equal to λ/2.45. Therefore, we could prove both numerically and experimentally that by reducing the periodicity and hole diameters, we can increase the resolution of same lens thickness.

## Discussion

In this work, we explored the higher bands of frequency for subwavelength imaging using resonant tunnelling. Primarily, we presented the layout of analytical evidence and then bolstered it with numerical and experimental data. Some inconsistencies were found among analytical, numerical, and experimental data. However, it was observed that by increasing the periodicity, the difference between analytical and numerical data tends to decrease. Furthermore, the difference in experimental data probably arose from the imperfection in coupling between two crystals which was demonstrated once. However, the data obtained were close enough to point out the fact that for a certain number of crystals, much higher band of frequencies exist, which was not found earlier. Moreover, we showed the effect of periodicity that with decreasing periodicity and consequently the hole diameters, the resolution can be increased. Furthermore, it was also found that the resonant tunnelling method has the advantage over FP resonance-based lens that the periodicity and hole diameter need not be much smaller than the wavelength of imaging frequency. Therefore, we expect our finding will draw more attention to resonant tunnelling method for its working capability in higher frequency regions and intrigue the research with it on ultrasonic subwavelength imaging for medical and non-destructive testing area.

## Methods

### Numerical Simulation

All the numerical simulations were conducted by using COMSOL multiphysics software, which is based on the Finite Element Method (FEM). Unit cells of 30 × 5 size were used to make a single crystal for the lens with periodicity (Λ) 5 and 6 mm; the results are shown in Figs 5, 7(c,d,e). For lens with periodicity Λ = 2.5 mm, 30 × 10 unit cells were used; the results can be seen in Fig. 7(f,g). Each lens contains two single crystal i.e. N = 2 and for every simulation, the sources were positioned 1 mm in front of the lens; the data were acquired 1 mm behind the lens.

### Experimental Data Processing

The data of Figs 6c,e and S6 were obtained with the speakers with diameter of 22 mm and Figs 6d and 7h,i with MEMS speakers with 1.5 mm diameter of its opening. The sound wave was generated by exciting the speakers with an Agilent 33522 A function generator. 10V_p-p_ and 2V_p-p_ was supplied via the function generator to the 22-mm speaker and MEMS speaker, respectively for this purpose. An (1/4″) G.R.A.S Type 26 CB microphone was placed behind the lens at a distance of 1 mm. A National Instruments DAQ (M/N:c DAQ-9171) card was used to convert the analog signal of the microphone to digital signal. Finally, a LabVIEW based software ‘NI Signal Express 2013’ was used for data processing. The output signal was acquired in DB for which the reference pressure was 20 µPa. Therefore, we converted the gain output into µPa using the formula, DB = 20 log(P/P_ref_). Then, all data were normalized.

## Electronic supplementary material

Supplementary Information
