# Supplementary material for: In-depth study on resonant tunneling for subwavelength imaging
Source: Sci Rep. 2018 Oct 19;8:15535. doi: 10.1038/s41598-018-33653-y (PMC6195579; doi:10.1038/s41598-018-33653-y)
Supplement: Supplementary file 1 — Supplementary Information [file 41598_2018_33653_MOESM1_ESM.docx]

**In-depth study on resonant tunneling for subwavelength imaging**

Md.Anzan-Uz-Zaman^1,2^, Kyungjun Song^1^, EunJoong Lee^1^ & Shin Hur^1,2,*^

^1^Department of Nature-Inspired Nano Convergence Systems, Korea Institute of Machinery and Materials, Daejeon, (34103) 156, the Republic of Korea

‎^2^Nano-Mechatronics,University of Science and Technology, Daejeon, (34113) 217, the Republic of Korea

^*^corresponding author: [shur@kimm.re.kr](mailto:shur@kimm.re.kr)

|   Y (mm)  Normalized Pressure |
| --- |
| **Figure S-1.** A part of the first band of second definite region. The distance between the sources 2Λ=12mm. |

|   Normalized Pressure  Y (mm) |
| --- |
| **Figure S-2.** A part of the first band of second definite region. The distance between the sources 2Λ=12mm. |

|   Normalized Pressure  Y (mm) |
| --- |
| **Figure S-3.** A part of the second band of second definite region. The distance between the sources 2Λ=12mm. |
|   Normalized Pressure  Y (mm) |
| **Figure S-4.** A part of the second band of second definite region. The distance between the sources 2Λ=12mm. |

|   Normalized Pressure  Y (mm) |
| --- |
| **Figure S-5.** Third band of second definite region. The distance between the sources 2Λ=12mm. |

|   Normalized Pressure  Y (mm) |
| --- |
| **Figure S-6.** Experimental study of second definite region to differentiate between imaging and non-imaging region. The thick solid lines represents imaging and thin lines non imaging region. The distance between the sources 4Λ=24mm. |

|   (a)  $k_{\parallel} \left( \mathrm{mm} \right)^{-1}$  Frequency (Hz)  $\left\vert T \right\vert$  Frequency (Hz)  1852  $\leq2.00$ |   (b)  $k_{\parallel} \left( \mathrm{mm} \right)^{-1}$  $\left\vert T \right\vert$  $\leq2.00$ |
| --- | --- |
| **Figure S-7.** Contour plots with same parameter as Fig. 3, except that the diameter of the unit cells were swapped. Crystal parameters: s = 2s_1_+s_2_ =20 mm, s_2_=2s_1_=10 mm, 4d_1_=d_2_=4 mm, and Λ=6 mm. **(a)** $\left\vert T \right\vert$ of the first definite region( 0‒2670 Hz); **(b)** $\left\vert T \right\vert$ of the second definte region (14480‒19820 Hz = 5340 Hz). | |

|   (b)  (a)  d=24mm  Normalized Pressure |   d=24mm  Normalized Pressure |
| --- | --- |
|   (c)  Normalized Pressure | |
| **Figure S-8. Simulation Result:** Normalized pressure amplitudes 1 mm behind the lens. The distance between the sources (d) is 24 mm. **(a)** Frequency at the first definite region for which subwavelength imaging was obtained. **(b)** New imaging frequency at the first definite region due to the exchange of diameters **(c)** No imaging is observed at second definite region. The portion of the BW is same as Fig.5-b where imaging was observed earlier with d_1_=4d_2_=4 mm. | |
